# Boron Doped Diamond Electrodes in Flow-Based Systems

**DOI:** 10.3389/fchem.2019.00190

**Published:** 2019-04-03

**Authors:** Jhonys Machado Freitas, Thiago da Costa Oliveira, Rodrigo Alejandro Abarza Munoz, Eduardo Mathias Richter

**Affiliations:** Institute of Chemistry, Federal University of Uberlandia, Uberlandia, Brazil

**Keywords:** batch injection analysis, capillary electrophoresis, flow injection analysis, liquid chromatography, review

## Abstract

Boron-doped diamond (BDD) electrodes present several notable properties, such as the largest potential window of all electrode materials (especially in anodic potentials), low background and capacitive currents, reduced fouling compared to other electrodes, mechanical robustness, and good stability over time. On the other hand, flow-based systems are known as well-established approaches to minimize reagent consumption and waste generation and with good compromise between sample throughput and analytical performance (mechanization of chemical assays). This review focuses on the use of BDD electrodes for electrochemical detection in flow systems, such as flow injection analysis (FIA), batch injection analysis (BIA), high performance liquid chromatography (HPLC), and capillary electrophoresis (CE). The discussion deals with the historical evolution of BDD, types of electrochemical pre-treatments (cathodically/H-terminated or anodically/O-terminated), cell configurations, and analytical performance. Articles are discussed in chronological order and subdivided according to the type of flow system: FIA, BIA, HPLC, and CE.

## Introduction

The attractive chemical and physical properties (wide potential window, low background currents, chemical and mechanical stability, good resistance to fouling, lack of a surface oxide film, and controllable surface termination) (Einaga, 2010) of boron-doped diamond (BDD) electrodes has been successfully exploited for multiple applications, such as in electrochemistry, analytical chemistry, electrocatalysis, environmental science (wastewater treatment and water disinfection/purification), biomedical or biological science, and other related trends (Luong et al., 2009; Macpherson, 2015).

Despite these attractive features, BDD is a semi-conductor doped material and usually is not considered as a typical electrode material for electrochemical applications (e.g., Pt, Au, and GC) (Macpherson, 2015). Therefore, some points should be considered when this material is used as the working electrode for electroanalytical applications: (i) usually, thin BDD films deposited on conductive substrates are used, and therefore, the surface cleaning procedures cannot be physical (e.g., mechanical polishing); (ii) the surface cleaning or pre-treatment more used and simpler is the electrochemical, such as anodic (O-terminated surface) or cathodic (H-terminated surface). This pre-treatment contributes significantly to the physical and chemical properties of the BDD and hence is of great importance for electroanalysis (often recommended to do daily for better reproducibility); (iii) different BDD growth face morphologies are commercially available and setting the best for each case is complicated; (iv) a recognized format for BDD electrodes has not currently widely available and alternative house made formats are typically adopted; (v) in electrochemical pre-treatments (anodic and cathodic) of BDD electrodes, high currents (±200 mA cm^−2^) or high potentials (> ±2 V) (Brocenschi et al., 2016) are often used, however, the adhesive materials (PDMS mounts, Kapton tape or insulating epoxy) commonly used for to define the geometrical electrode area may not be resistant to this pre-treatment and noticeable decrease in performance (increase in background current) and in reproducibility (variation in the geometric area) can be observed.

The purpose of this review is to outline the state of knowledge and discuss the advantages and disadvantages related to the use of BDD electrodes in electrochemical detection coupled to flow systems: flow injection analysis (FIA), batch injection analysis (BIA), high performance liquid chromatography (HPLC), and capillary electrophoresis (CE).

## Fabrication and Surface Activation Procedures for BDD Electrodes

Natural or undoped diamond is an electrical insulator (resistivity ≈ 10^20^ Ω·cm) and cannot be used as an electrode material. However, after p-doped with boron (usual between 10^18^ and 10^21^ atom cm^−3^), a decrease in resistivity is achieved (<1 Ω·cm) and then diamond films can be used as electrode materials for diverse electrochemical applications (Luong et al., 2009). The effect of boron doping levels on the electrochemical properties of BDD electrodes has been extensively studied (Bogdanowicz et al., 2013). From the point of view of electroanalysis, BDD electrodes with boron doping levels of around 10^20^ boron atoms per cm^−3^ (2,000–8,000 ppm) can be used successfully. The absence of sp^2^ carbon impurities (graphite) is an important characteristic to be considered, since the presence of this impurity increases the background current and decreases the potential window (Watanabe et al., 2010). Most of the BDD films employed as electrodes are produced by chemical vapor deposition (CVD). This is due to efficiency of the process in controlling dopant incorporation and the capacity to grow over large areas on structured conductive bases (Macpherson, 2015). The presence of hydrogen radicals during the CVD process is essential because it selectively prevents the formation of non-diamond carbon (graphite) on the diamond surface. Two methods of activation are dominant in the field: hot filaments or microwave reactors (Butler et al., 2009). The gas mixture that supplies the reaction chamber usually is compounded by methane (as carbon source) dispersed in a large amount of hydrogen (radical source and carrier gas) (Pleskov et al., 1987; Tokuda, 2015). The use of a mixture of acetone and methanol as carbon source were also reported (Yano, 1998; Rao et al., 2000; Ivandini et al., 2002, 2007). As boron source, B_2_O_3_ or trimethyl-boron are frequently used. The structure and the deposition rate of the BDD film depends on the technique used, generally, processes that employed plasma techniques are faster than those employing hot filament. However, the latter is more convenient for the deposition of doped diamond on large surfaces (Luong et al., 2009). Different types of substrates can be used for deposition of BDD films, such as silicon (Haenni et al., 1998), tungsten (Pleskov et al., 1987), and niobium (Hayashi et al., 2012), among others (Luong et al., 2009). Silicon substrates are the most used bases in electroanalysis. The convenient experimental conditions, such as temperature, pressure, gas composition, as well as, the procedures to characterization of the films produced are presented in detail elsewhere (Yano, 1998; Martin et al., 1999; Luong et al., 2009).

The electrochemical properties of BDD thin-film electrodes can be altered through different procedures, such as boiled in strong acid (Hayashi et al., 1997), by anodic or cathodic polarization (Granger and Swain, 1999; Goeting et al., 2000; Zhang et al., 2004), hydrogen plasma treatment (Granger and Swain, 1999), and also by long-term exposure to air (Kulesza et al., 2004). Therefore, when BDD electrodes are used, special care should be adopted to achieve the best electrochemical performance with this material. The physical, chemical and electronic characteristics of BDD surfaces, and consequently its electrochemical response, are strongly influenced by the BDD surface termination. Hydrogen-terminated surfaces present a negative electron affinity and a high conductivity, whereas oxygen-terminated ones are hydrophilic with a positive electron affinity and have a low conductivity (Salazar-Banda et al., 2006). When BDD electrodes are coupled to flow cells, the surface termination is usually generated by electrochemical methods: (i) hydrogen evolution by cathodic pre-treatment to produce H-termination or (ii) oxygen evolution by anodic pre-treatment to produce O-termination. However, it is important to emphasize that the electrochemical pre-treatment must be constantly remade (usually once a day) because hydrogen terminations can be converted to oxygen terminations if higher anodic potentials are used (> +1.2 V) (Oliveira and Oliveira-Brett, 2010). It is also important to emphasize that the time of application of the electrochemical pre-treatment depends on the previous use of the BDD electrode. Conditions, such as time of exposure to air, applied potential, and type of analyte under study or analysis generate different modifications on the BDD surface. Finally, it is important to emphasize that the H-terminated BBD surface cannot be fully retrieved by cathodic pre-treatments, even if severe electrochemical pretreatments were used (high potentials and longer times). However, a partial recovery (15%) is usually enough to ensure reversible voltammetric behavior for the model molecule Fe(CN)_6_]^3−/4−^ (Brocenschi et al., 2016). The use of a redox specie as a secondary indicator level (e.g., ascorbic acid, which is sensitive to the hydrogenation) can be an interesting strategy to have quick information about of the relative atomic bonding structure on the BDD surface after electrochemical pre-treatment without the use of X-ray photoelectron spectroscopy analysis (Brocenschi et al., 2016).

## Flow Cell Configurations

**Figure 1:**
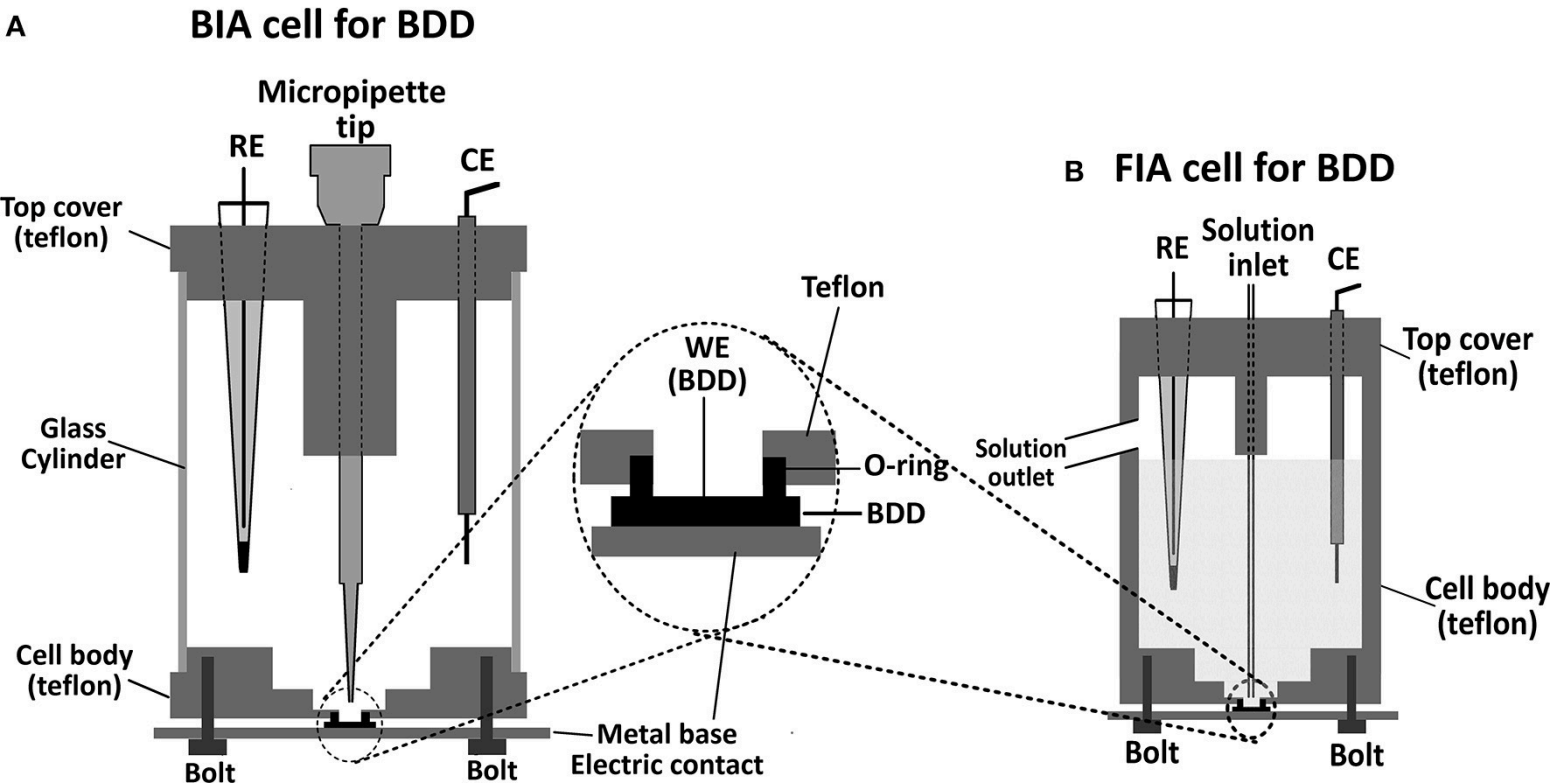
**(A)** Schematic diagram of batch injection analysis (BIA) and **(B)** flow injection analysis (FIA) cells for BDD electrodes.

In the construction of a flow-cell for electrochemical detection, the type and characteristics of the material which will be used as the working electrode should be considered. In the case of BDD electrodes, reproducible results are usually obtained if an electrochemical cleaning or pre-treatment (anodic and/or cathodic) is carried out routinely. When the pre-treatment is carried out, the evolution of air bubbles (O_2_ or H_2_) is usually intense on the BBD surface and the electrical contact between the electrodes can be easily interrupted in a common flow cell, and so, the electrochemical pre-treatment of the BBD electrode is not possible with the electrode coupled to the flow cell. One alternative is to make the electrochemical pre-treatment of BDD electrodes before positioning in a flow cell arrangement (Andrade et al., 2009; Santos et al., in press). However, this procedure (pretreatment in one cell and then positioning in another) is a tedious and laborious task, even if adequate manpower and laboratory facilities are available. An efficient and useful way to define the area of BDD electrodes was shown by Granger et al. (2000). The BDD thin film grown on a conductive silicon substrate was pressed against the bottom of the glass cell with the fluid being contained by a Viton or rubber O-ring. A few years later, this strategy was used for positioning of BDD electrodes in wall-jet flow cells, both for use in FIA (Silva et al., 2011) or BIA (Tormin et al., 2011) systems. Both cells are very similar, only with differences in the volume of the solution into the cells, as can be seen in Figures 1A (BIA), 1B (FIA). If the internal volume of a BIA cell is large, the dilution factor is great, and many analyses (usually more than 200 injections) are possible without exchange the supporting electrolyte inside the cell (useful in field analysis). A great advantage of this configuration is that the electrochemical cleaning or pre-treatment (anodic and/or cathodic) of the BDD electrode can be easily performed with the electrode positioned in the flow cell. Despite the advantages of wall-jet cells if BDD electrodes are used, thin-layer flow cells (Maixnerová et al., 2012) can also be used, however, is this case, the electrochemical pretreatment has restrictions in this type of cell configuration.

## BDD Electrode Coupled to FIA Systems

First proposed by Ṙužič and Hansen (1975), flow injection analysis (FIA) is considered as an analytical technique where the analytical signal is based on quick introduction of a small volume of sample into a turbulently flowing carrier stream of reagent, on-line sample processing (carried out to provide/enhance selectivity of detector response), and detection during the flow of the analyte (in non-derivatized or chemically derivatized form) through the system detector (Trojanowicz, 2011). Various types of detection modes are applied to FIA, such as spectrophotometry (notably UV-Vis), luminescence, atomic spectroscopy and electrochemical detection (amperometry, potentiometry, conductometry). When electrochemical methods are used, features like the versatility of the detector design, high sensitivity and wide linear concentration range meet most of the requirements of flow analysis (Siangproh et al., 2009). The flow rate can be either parallel to the electrode surface (thin-layer cells) or perpendicular to the electrode surface (wall-jet cells).

BDD electrodes were firstly employed as detectors in flow systems in 1997, by Jolley et al. (1997). In this study, the amperometric detection of ferrocyanide, ferricyanide, ethylamine, and ethylenediamine using bare BDDE (without any superficial pre-treatment) was investigated for the first time. The BDD performance was compared against glassy carbon electrode (GCE) and evaluated as a function of characteristics, such as linear range, sensitivity, limit of detection, and stability. In this work, the authors concluded that diamond films leaded to improved response stability and S/B ratios as compared with those obtained with polished glassy carbon. Two years later, Granger et al. (1999) published another paper showing the amperometric oxidation of chlorpromazine, ascorbic acid, dopamine and 4-methycatecholamine using BDD coupled to the FIA system. In this study, the performance of BDD electrodes presented lower LODs and noteworthy fouling resistance compared with the GCE. The same paper also presents results for analytes, such as azide and nitrite, separated by ion chromatography. A few FIA-AMP methods also demonstrates the oxidation of the neurotransmitters, histamine, serotonin and its metabolite, 5-hydroxyindoleacetic acid (Sarada et al., 2000), polyamines (i.e., cadaverine, putrescine, spermine, spermidine) (Witek and Swain, 2001) and glutathione and cephalexin (Hailapakul et al., 2001) in high-quality, boron-doped diamond thin film electrodes. The performance of the diamond electrode is superior than that presented by GCE in these articles, since the background current and the adsorption of the organic compounds on the surface of the diamond electrode is lower than that presented by the CGE. However, these studies aimed at the application of BDD electrodes as detectors on FIA systems using various compounds as model analytes and no analysis of real samples was performed.

In the following years, Wangfuengkanagul and Chailapakul (Wangfuengkanagul and Chailapakul, 2002a) firtsly applied the BDD electrode coupled to a FIA system for the quantification of acetaminophen in pharmaceutical samples. The performance of the BDD electrode was evaluated through various validation parameters (linearity, accuracy, recovery, precision, electrode stability). The FIA results were obtained with a reproducible response (RSD < 2.3% without electrode pretreatment or electrode modification or using additional potential pulse for electrode cleaning). Linear dynamic range was obtained over two orders of magnitude (from 0.5 to 50 μM) and the LOD for acetaminophen detection was 10 nM. Similar results were reached by the same authors for the analysis of D-penicillamine (with BDD electrodes showing significant advantages over the GCE, RSD < 2.1%, and recovery values between 98 and 102%) (Wangfuengkanagul and Chailapakul, 2002b). However, for the analysis of four tetracyclines by FIA, Wangfuengkanagul et al. (2004) showed that the electrochemical pretreatment of the BDD surface is useful to enhance the voltammetric response obtained for the oxidation of the tetracyclines (tetracycline, chlortetracycline, oxytetracycline, and doxycycline. The electrochemical pretreatment proposed in this work was the scanning from 0.0 to 2.2 V (vs. Ag/AgCl) using cyclic voltammetry (40 cycles) in 0.1 M KOH solution. The limit of detection was 10 nM for all tetracyclines.

The excellent performance of BDD electrodes in FIA systems with amperometric detection was explored by several investigators for determination of numerous organic compounds in pharmaceutical samples, such as sodium thiosulphate (Suryanarayanan et al., 2004), malachite green and leucomalachite green (Ngamukot et al., 2006), bovine serum albumin and immunosuppressive acidic protein (Chiku et al., 2008), oxalate (Kondo et al., 2008), captopril (Siangproh et al., 2003a), tiopronin (Siangproh et al., 2003b), lincomycin (Boonsong et al., 2005), chloramphenicol (Chuanuwatanakul et al., 2008), nimesulide (Lima et al., 2013), yohimbine (Švorc and Kalcher, 2014), N-acetyl-l-cysteine (Nantaphol et al., 2014), hydrazine (Channon et al., 2015), and both epinephrine and acetaminophen (Lourenção et al., 2017). Besides organic compounds, BDD electrode coupled to FIA-AMP systems was successfully applied for quantification of iodide contents in nuclear emergency tablets (Chailapakul et al., 2004). Information's about type of electrochemical pre-treatment, limit of detection, linear range, and analytical frequency can be found in Table 1.

**Table 1:** FIA systems with electrochemical detection using BDD as the working electrode.

**DT**	**Pre-treatment**	**Analyte**	**Sample**	**LOD (μM)**	**LR (μM)**	**AF (h^**−1**^)**	**References**
AMP	As received	Ethylamine (EA) Ethylenediamine (EDA)	SS	5 (EA and EDA)	10–1,000 (EA) 10–1,000 (EDA)	40	Jolley et al., 1997
AMP	Hydrogen plasma (800°C/30 min)	Chlorpromazine (CPM) Ascorbic Acid (AA) Dopamine (DP) 4-Methylcathecol (4-MC)	SS	0.004 (CPM) 0.012 (AA) 0.002 (DP) 0.002 (4-MC)	0.3–3,000 (CPM) 0.1–3,000 (AA, DP and 4-MC)	NR	Granger et al., 1999
AMP	NR	Histamine (HIS) Serotonine (SER) 5-Hydroxyindoleacetic acid (HIAA)	SS	0.5 (HIS) 0.02 (SER) 0.1 (HIAA)	0.5–100 (HIS) 0.01–50 (SER) 0.1–100 (HIAA)	40	Sarada et al., 2000
AMP	As received	Cadaverine (CAD) Putrescine (PUT) Spermine (SPR) Spermidine (SPD)	SS	1.0(CAD/PUT/SPD) 0.3 (SPR)	1.0–1,000 (CAD, PUT and SPD) 0.3–1,000 (SPR)	NR	Witek and Swain, 2001
AMP	As received	Glutathione (GLU) Cephalexin (CPH)	NR	0.5 (GLU)	0.5–100 (GLU)	NR	Hailapakul et al., 2001
AMP	As received	Acetaminophen	PF	0.01	0.5–50	76	Wangfuengkanagul and Chailapakul, 2002a
AMP	+3.0 V/30 min 0.1 M H_2_SO_4_	Bisphenol-A 17β-estradiol	NR	1 10	1–100	30	Notsu et al., 2002
AMP	As received	D-penicillamine	PF	0.01	0.5–50	54	Wangfuengkanagul and Chailapakul, 2002b
AMP	As received	Captopril	PF	0.01	0.5–100	35	Siangproh et al., 2003a
AMP	As received	Tiopronin	SS and PF	0.01	0.5–50	NR	Siangproh et al., 2003b
AMP	Cyclic voltammetry (0–2.2 V)/30 min/0.1 M KOH	Tetracycline Chlortetracycline Oxytetracycline Doxycycline	PF	0.01	0.5–50	NR	Wangfuengkanagul et al., 2004
AMP	As received	Iodide ion	PF	0.01	0.8–200	85	Chailapakul et al., 2004
AMP	As received	Sodium thiosulphate	SS	0.2	0.5–100	180	Suryanarayanan et al., 2004
AMP	NR	Glucose	SS	0.67 (Ni-BDD) 10 (Cu-BDD)	1–50	NR	Ivandini et al., 2004
AMP	As received	Lincomycin	PF	0.02	0.5–125	90	Boonsong et al., 2005
AMP	NR	Hydrogen peroxide	SS	0.05	0.1–10	NR	Ivandini et al., 2005
AMP	As received	Tetracycline	PF	0.01	1–100	NR	Treetepvijit et al., 2005
AMP	−3.0 V/30 min/0.1 M BR buffer, pH = 5.0	Rutin	Tea samples	7.7	10–250	100	Pedrosa et al., 2006
PAD	As received	Salbutamol (SBM) Terbutaline (TBL) Clenbuterol (CBR)	PF	0.1 (SMB) 0.5 (TBL) 0.3 (CBR)	0.5–100 (SBM) 1.0–100 (TBL) 0.5–50 (CBR)	NR	Karuwan et al., 2006
AMP	As received	Malachite green (MG) Leucomalachite green (LMG)	SS	0.05 for both	1–100 (MC) 4.0–80 (LMC)	144	Ngamukot et al., 2006
AMP	As received	Sulfonamides	Eggs	0.04 (SDZ) 0.04 (SMZ) 0.04 (SMM) 0.10 (SDM)	0.2–400 (SDZ) 0.18–359 (SMZ) 0.18–357 SMM) 0.32–967 (SDM)	NR	Preechaworapun et al., 2006
AMP	As received	Arsenite (As(III))	Tap water	0.02	0.1–100	30	Ivandini et al., 2006
AMP	As received	Chloramphenicol	PF/Milk samples	0.03	0.1–50	60	Chuanuwatanakul et al., 2008
AMP	−3 V/10/0.1 M PBS pH 7.4.	Bovine serum albumin (BSA) Immunosuppressive acidic protein (IAP)	SS	0.76 for BSA2.0 for IAP	0.76–455 BSA 4.0–16.0 (IAP)	48	Chiku et al., 2008
AMP	As received	Oxalate	SS	0.03	0.8–100.0	23	Kondo et al., 2008
MPA	−1.0 A cm^−2^/120 s/0.5 M H_2_SO_4_	BHA BHT	Food samples	0.03 (BHA) 0.4 (BHT)	0.05–3.0 (BHA) 0.7–70 (BHT)	63	Medeiros et al., 2010b
MPA	−3.0 V/900 s/0.5 M H_2_SO_4_	Paracetamol (PAR) Caffeine (CAF)	PF	0.7 (PAR) 0.9 (CAF)	53–1,300 for PAR 5.1–132 for CAF	140	Silva et al., 2011
MPA	3.0 V/900 s/0.5 M H_2_SO_4_	Diclofenac (DCF)	PF	0.1	5–50.0	135	Gimenes et al., 2011
MPA	−9 C cm^−2^ 0.5 M H_2_SO_4_	Sildenafil citrate	PF	0.4 0.04	2–100 0.6–100	86	Lopes Júnior et al., 2012
MPA	0.5 A cm^−2^/60 s and −0.5 A cm^−2^ for 180 s/0.5 M H_2_SO_4_	Tartrazine and sunset yellow (TT–SY)	Food samples	2.5 (TT) 0.8 (SY)	5–60 (TT) 1–50 (SY)	80	Medeiros et al., 2012
		Brilliant blue and SY (BB–SY)		3.5 (BB) 0.9 (SY)	5–60 (BB) 1–50 (SY)		
AMP	+11.7 mA/30 s/0.5 M H_2_SO_4_	Nimesulide	PF	0.08	4–80	90	Pereira et al., 2013
AMP	−2.0 V/60 s/0.5 M H_2_SO_4_	Yohimbine	Dietary supplements	0.2	0.3–10 10–100	70	Švorc and Kalcher, 2014
FBA-DPASV	−50 mA cm^−2^/120 s/0.1 M H_2_SO_4_.	Zn^2+^ Pb^2+^	Water samples	9.2 × 10^−3^ (Zn^2+^) 9.6 × 10^−4^ (Pb^2+^)	0.18–1.23 (Zn^2+^) 3.1 × 10^−3^ −5.2 × 10^−2^ (Pb^2+^)	12	Bezerra dos Santos et al., 2014
MCFA–MPA				3.4 (Zn^2+^) 0.2 (Pb^2+^)	12.7–76.2 (Zn^2+^) 0.2–3.9 (Pb^2+^)	67	
AMP	As received	Hydrogen Sulfide	Aqueous solutions	0.4	1–51	24	Bitziou et al., 2014
AMP	As received	N-acetyl-l-cysteine (NAC)	PF	0.01	0.5–50	64	Nantaphol et al., 2014
AMP	−0.5 A cm^−2^/360 s/0.50 M H_2_SO_4_	Estrone 17-β-estradiol Estriol	Tap and natural waters	0.01	0.1–3.0	50	Brocenschi et al., 2014
SWASV	50 mA cm^−2^/120 s/0.1 M H_2_SO_4_	Cd^2+^ Pb^2+^	Natural waters	1.6 × 10^−3^ (Cd^2+^) 3.9 × 10^−4^ (Pb^2+^)	0.06–0.45 (Cd^2+^) 0.01–0.11 (Pb^2+^)	NR	Bezerra dos Santos et al., 2015
MPA	−0.01 A/120 s/0.5 M H_2_SO_4_	Caffeine (CAF) Ibuprofen (IB) Paracetamol (PC)	PF	0.2 (PC) 0.2 (CAF) 0.1 (IB)	14–281 (PC) 3–60 (CAF) 10–205 (IB)	150	Chaves et al., 2015
MPA	0.5 A cm^−2^/180 s/0.5 M H_2_SO_4_	Hydrochlorothiazide (HTZ) Enalapril (ENP)	PF	0.2 (HTZ) 0.01 (ENP)	0.4–8.0 (HTZ) 0.03–1.00 (ENP)	89	Lourenção et al., 2015
AMP	Polished with alumina slurry (0.05 μm)	Hydrazine (HZ)	PF	64.5	1–100	NR	Channon et al., 2015
MPA	−0.04 A cm^−2^/180 s/0.5 M H_2_SO_4_	Acetaminophen (ACP) Tramadol (TRA)	PF and BF	0.03 (ACP) 0.04 (TRA)	1–100 (ACP) 0.08–10 (TRA)	85	Santos et al., 2015
MPA	−0.03 A/360 s/0.5 M H_2_SO_4_	Cotinine (CO)	BF	0.06	0.5–100.0	36	Alecrim et al., 2016
AMP	−2.0 V/180 s/1 M H_2_SO_4_	Ziram (pesticide)	Natural water	0.003	0.1–1	6	Stanković and Kalcher, 2016
MPA	−0.03 A/360 s/0.5 M H_2_SO_4_	Prazosin	PF	0.5	2–200	70	Guedes et al., 2016
AMP	+3.0 V/20 min phosphate buffer (pH 7.4)	Oxytocin and Vasopressin	BF	0.05	0.1–10	60	Asai et al., 2016
AMP	As received	Oxalic acid	Natural water	NR	10–100	NR	Watanabe et al., 2016
AMP	0.5 A cm^−2^/60 s and −0.5 A cm^−2^/180 s/0.5 M H_2_SO_4_	Ivermectin (IVM) and Levamisole (LVM)		0.03 (IVM) 0.001 (LVM)	0.6–50.0 (IVM) 0.01–5.0 (LVM)	48 (IVM) 56 (LVM)	Lourenção et al., 2016
MPA	−0.04 A cm^−2^/180 s/0.5 M H_2_SO_4_	Acetaminophen (ACM) Codeine (COD)	PF and BF	0.03 (ACM) 0.04 (COD)	0.08–100 (ACM) 0.05–10 (COD)	90	Santos et al., in press
AMP	As received	Epinephrine (EP) and Acetaminophen (AC)	PF	0.5 (EP) 0.7 (AC)	0.6–30.0 (EP) 0.8–70.0 (AC)	45 (EP) 50 (AC)	Lourenção et al., 2017
MPA	+1 mA/120 s and, −30 mA/360 s/0.5 M H_2_SO_4_	Warfarin	PF	0.5	2–400	94	de Jesus Guedes et al., 2017
MPA	−0.10 A cm^−2^/120 s/0.5 M H_2_SO_4_	Indigo carmine Allura red	Candy samples	0.04 0.007	0.07–1.0 0.04–0.7	153	Deroco et al., 2018
MPA	−0.03 A/360 s/0.1 M H_2_SO_4_	Colchicine	PF and BF	0.02	0.1–2.0 20–500	30	Moreira et al., 2018
MPA	0.03 A/360 s/0.1 M H_2_SO_4_	Verapamil	PF and BF	0.2	0.8–40	45	Barbosa Lima et al., 2018
AMP	−2.9 V/340 s/3 M H_2_SO_4_	Hydrogen peroxide	Tooth gel	1.1	9.8–95.9	63	Azevedo et al., 2018
MPA	−30 mA/360 s/0.5 M H_2_SO_4_;	Oxcarbezepine	PF and BF	0.4	2–80	65	Lima et al., 2018

*DT, detection technique; SS, standard solution; PF, pharmaceutical formulations; BF, biological Fluids; PAD, pulsed amperometric detection; AMP, chronoamperometric detection; MPA, multiple pulse amperometry detection; SWASV, square-wave anodic stripping voltammetry detection; FBA-DPASV, flow-batch analysis with differential pulse anodic stripping voltammetry detection; MCFA–MPA, multicommutation flow analysis with multiple pulse amperometry detection; LOD, limit of detection; AF, analytical frequency; LR, linear range; NR, not reported*.

In addition to pharmaceutical preparations, samples, such as eggs, tea, milk, and natural water were also analyzed using BBD electrodes coupled to FIA systems with chronoamperometric detection. In this sense, Preechaworapun et al. (2006) analyzed hen egg samples to detect four sulfonamides: sulfadiazine (SDZ), sulfamethazine (SMZ), sulfamonomethoxine (SMM), and sulfadimethoxine (SDM). The method presented linear range from 0.50 to 100 ppm for SDZ/SMZ/SMM and from 0.100 to 300 ppm for SDM; the obtained LODs were 0.011 ppm and 0.032 ppm for SDZ and SDM, respectively. The percentage of recoveries was found to be between 90 and 108%. Rutin, a phenolic derivate compound, was analyzed in tea samples by Pedrosa et al. (2006) using a cathodically pretreated BDD electrode (30 min at −3.0 V in 0.1 M BR buffer, pH = 5.0). The method presented linear dynamic range from 10 to 250 μM, LOD of 7.7 μM (three times lower than the value calculated for the CGE) and high analytical frequency (240 samples h^−1^). In other study, Chuanuwatanakul et al. (2008) used a bare BDD electrode for the determination of chloramphenicol in sterile eye drops and milk samples. The method presented a large linear dynamic range (0.05–1,000 μM), LOD of 0.03 μM and relative standard deviations of 3.5 and 3.0% for 5 and 10 μM (*n* = 10) of chloramphenicol, respectively. These results were obtained even without any surface pretreatment of the BDD electrode. Endocrine-disrupting compounds, most notably estriol, 17-β-estradiol and estrone were analyzed in tap and natural waters using a cathodically pretreated BDD (Brocenschi et al., 2014). For all three compounds, the method presented linear response for concentration range from 0.10 to 3.0 μM and LOD of 0.10 μM. The authors also mention that the cathodically pretreated BDD electrode presents less surface fouling when compared to GCE mainly because its hydrogen-terminated surface (non-polar character) and absence of an extended π-electron system.

Alternatively, other FIA methods with electrochemical detection were also implemented using BDD electrodes. In 2010, the simultaneous determination of antioxidants BHA and BHT was carried out in mayonnaise samples (Medeiros et al., 2010a). In this work, for the first time, a BDD electrode was used for simultaneous determination in a FIA system coupled with multiple pulse amperometric detection (FIA-MPA) (dos Santos et al., 2008; Santos et al., 2009). A dual-potential waveform [E_det1_ = +0.85 V and E_det2_ = +1.15 V (vs. Ag/AgCl)] was employed with acquisition of two separate amperograms. BHA was detected at +0.85 V without interference of BHT and at +1.15 V, both compounds (BHA and BHT) were detected. The selective determination of BHT was possible with the subtraction of the current detected at +0.85 V (i = i_+1.15V_ – i_+0.85V_). Very low detection limits were obtained for the simultaneous determination of BHA (0.03 μM) and BHT (0.40 μM), as well as high analytical frequency (80 injections h^−1^).

In 2011, Silva et al. (2011) used a FIA-MPA system for simultaneous determination of paracetamol (PAR) and caffeine (CAF) in pharmaceutical formulations. In this work, PAR was selectively detected at a potential pulse of +1.20 V, while both PAR and CA were detected at potential pulse of +1.55 V (vs. Ag/AgCl). A third potential pulse (+0.40 V) was applied in order to prevent superficial fouling of the BDD electrode during the analysis. Good analytical frequency was obtained (140 injections h^−1^) and the limits of detection were 0.66 μM for PAR and 0.87 μM for CAF. In this work, the use of the correction factor was proposed for the first time. This strategy made simultaneous determinations feasible in numerous samples containing more than one compound using a single working electrode and flow systems with amperometric detection. A similar approach was employed by Medeiros et al. (2012) for simultaneous determination of two pairs of food colorants: tartrazine and sunset yellow (TT–SY) or brilliant blue and SY (BB–SY). The detection limits were 2.5, 0.80, and 3.5 μM for TT, SY, and BB, respectively.

The advantages of FIA systems coupled to BDD electrodes and multiple pulse amperometric (MPA) detection was explored for simultaneous determinations of different compounds in subsequent years, such as caffeine, ibuprofen and paracetamol (Chaves et al., 2015), hydrochlorothiazide and enalapril (Lourenção et al., 2015), acetaminophen and tramadol (Santos et al., 2015), acetaminophen and codeine (Santos et al., in press), epinephrine and acetaminophen (Lourenção et al., 2017), and food dyes (indigo carmine and allure red) (Deroco et al., 2018). It is worth mention that all works employed a cathodically pretreated BDD electrode. FIA systems with pulsed amperometric detection (similar to MPA) using BDD as working electrode were also used for the determination of a single species in pharmaceutical formulations, as demonstrated for salbutamol, terbutaline, and clenbuterol (Karuwan et al., 2006), diclofenac (Gimenes et al., 2011), sildenafil citrate (Lopes Júnior et al., 2012), cotinine (Alecrim et al., 2016), prazosin (Guedes et al., 2016), warfarin (de Jesus Guedes et al., 2017), colchicine (Moreira et al., 2018), verapamil (Barbosa Lima et al., 2018), and oxcarbazepine (Lima et al., 2018). Colchicine, verapamil, and oxcarbazepine were also determined in biological fluids. The MPA technique allows the use of a simple strategy to obtain selectivity for determination of some compounds in complex samples (e.g., urine) based on the detection of a product generated in a previous potential pulse. This analysis would not be possible if the conventional amperometry is used due to the interferences of electroactive species in the sample matrix, e.g., ascorbic or uric acids. Some analytical characteristics of the aforementioned works can be found in Table 1.

**Figure 2:**
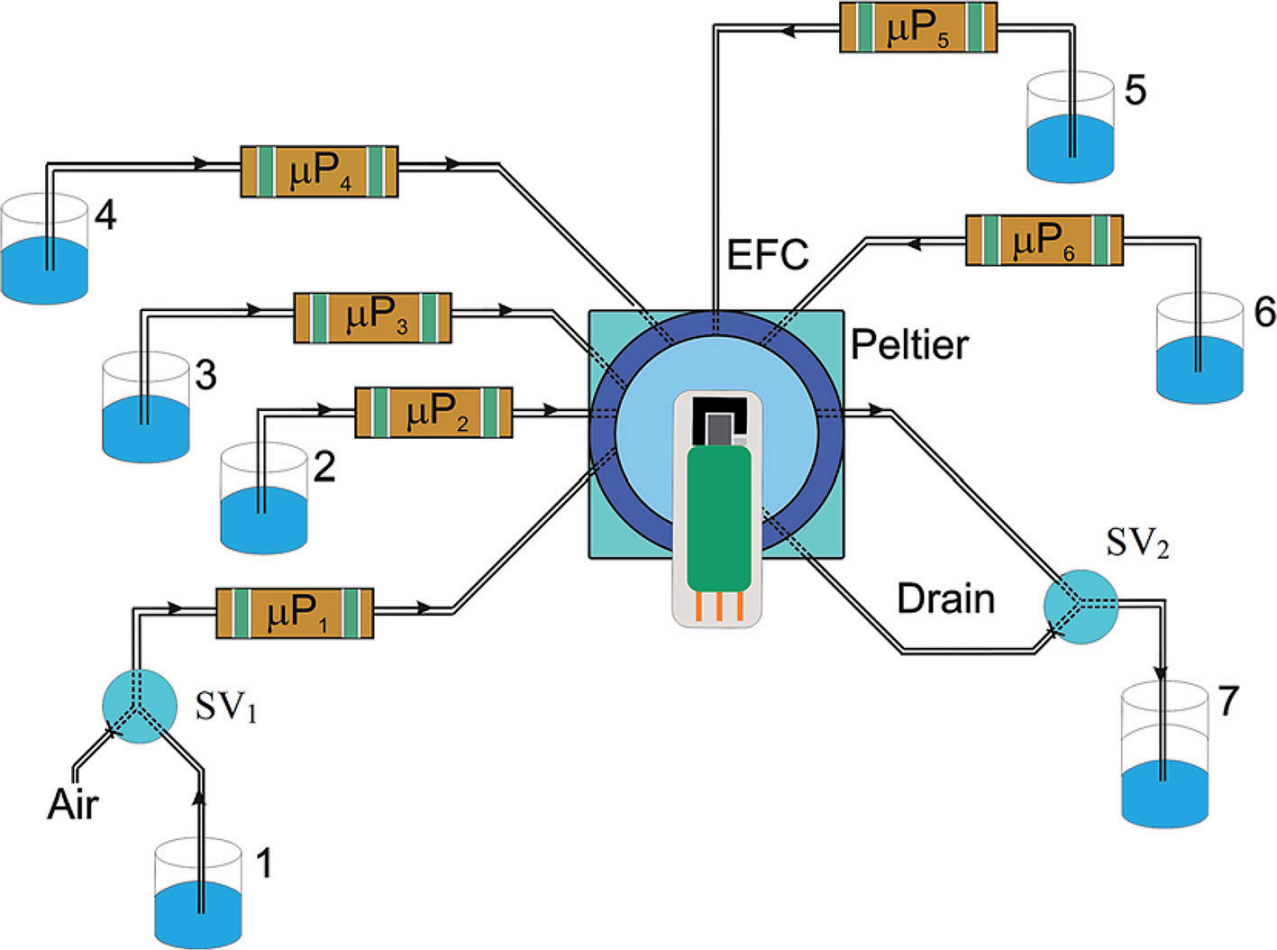
Flow manifold developed for FBA-DPASV and MCFA-MPA. The μP1 and μP5 were used to pump 50 μL per pulse of acetic acid/acetate solution at pH 4.0 (1) and 0.1 M H_2_SO_4_ (5), respectively. The μP2, μP3, μP4, and μP6 pump 20 μL per pulse of the stock solutions of Zn^2+^ (2), Pb^2+^ (3), interferents (4), and samples (6), respectively. SV1 and SV2 are the three-way valves and the waste (7), respectively. Reprinted from Bezerra dos Santos et al. (2014). Reproduced by permission of The Royal Society of Chemistry.

The BDD electrode coupled to FIA systems was also used for the simultaneous determination of Zn^2+^ and Pb^2+^. Two distinct flow electrochemical methods were proposed by Bezerra dos Santos et al. (2014) with the adaptation of the BDD electrode on an screen-printed electrode (SPE) design: (i) flow-batch analysis (FBA) with differential pulse anodic stripping voltammetric (DPASV) detection; and (ii) multicommutation flow analysis (MCFA) with multiple pulse amperometric (MPA) detection. The SPE–BDD device presented reduced dimension in order to be coupled to a miniaturized electrochemical flow cell. Figure 2 presents the flow manifold for on-line pretreatment of the SPE–BDD and its application to determine Zn^2+^ and Pb^2+^ ions.

The MCFA-MPA method presented LODs of 41 and 220 ppb for Pb^2+^ and Zn^2+^, respectively, which were 200 and 300 times higher, when compared to those obtained by FBA-DPASV (0.19 and 0.62 ppb, respectively). On the other hand, the MCFA-MPA system presented an analytical frequency six times higher than the FBA-DPASV. Shortly after, the same research group presented a study for *in situ* simultaneous determination of Cd^2+^ and Pb^2+^ in natural waters, employing a flow-batch analysis with square-wave anodic stripping voltammetric detection (FBA-SWASV) (Bezerra dos Santos et al., 2015). The developed electrochemical analyzer was capable of on-line data transmission and used a solar board as renewable energy source. The limits of detection were 0.08 ppb and 0.18 ppb for Pb^2+^ and Cd^2+^, respectively.

BDD electrodes modified with different metals (such as Pt, Ni, Ir, and Cu) were also used in FIA systems for the detection of a wide range of analytes. Ivandini et al. (2004) fabricated metal-modified BDD electrodes using the ion implantation method (750 keV Ni^2+^ and Cu^2+^ with a dose 5 × 10^14^ cm^−2^ using metal rods as target). These modified BDD electrodes exhibited high catalytic activity, excellent electrochemical stability and low background current for glucose oxidation in alkaline media. The small background current resulting in lower detection limit (670 nM) for glucose determination in comparison to those obtained with related metal electrodes. Shortly after, similar procedure was used to obtain Ni and Pt-modified BDDs for the detection of tetracycline (Treetepvijit et al., 2005) and hydrogen peroxide (Ivandini et al., 2005). Superior results in relation to sensitivity and stability have also been observed in comparison to those obtained with bare BDD and metal electrodes (Ni and Pt). In other work, similar procedure was used to produce Ir-implanted BDD electrodes which were exploited for the detection of As(III) (Ivandini et al., 2006). The ion implantation method offered more chemically, electrochemically, and physically stability if compared with the electrodeposition method for the modification of BDD electrodes. Superficial modification of BDD was also evaluated for the determination of estrogenic phenolic derivates (estrogenic derivatives, bisphenol-A, and 17β-estradiol), as reported by Notsu et al. (2002). In this work, BDD electrodes were firstly anodically polarized for the introduction of hydroxyl groups onto its surface, then treated with (3-aminopropyl)-triethoxysilane and finally modified with a tyrosinase film cross-linked with glutaraldehyde. The modified electrode was stable for more than 12 h during repeated injections of samples containing various concentrations and kinds of phenol derivatives and retained its initial sensitivity for a few days if storage under dry conditions. The limit of detection for bisphenol-A was 1 μM. Recently, the detection of acetaminophen and epinephrine has been shown using a porous BDD electrode grown by hot filament chemical vapor deposition over oxygen plasma functionalized carbon nanotubes (Lourenção et al., 2017). The limits of detection were 0.50 μM (epinephrine) and 0.70 μM (acetaminophen) and the method was successfully used to analysis of spiked serum samples (recovery from 101 to 110%).

## BDD Electrode Coupled to BIA Systems

Batch injection analysis (BIA) is a system conceptually comparable to FIA, because it is also based on reproducible transport of a solution toward a detector. Firstly presented in 1991 by Wang and Taha, the technique consists of injection of small sample aliquots (usually using an electronic pipette) directly on the detector surface (wall-jet arrangement), which is immersed in a large-volume of supporting electrolyte (blank solution) (Wang and Taha, 1991). The passage of the analyte over detector's surface generates a peak-shaped response, similar to that obtained in FIA systems. The magnitude of the peak is proportional to the concentration of the analyte in the injected solution (Wang and Taha, 1991; Quintino and Angnes, 2004). After injection of a microliter sample solution (10–150 μL) on an electrode surface immersed in a large-volume of supporting electrolyte, a transient peak-shaped response is observed due to the reproducible transport and/or controlled dispersion (high dilution) in space and time. The injection procedure can be carried out with the solution in the BIA cell under stirring (faster analysis) (Wang and Taha, 1991; Pereira et al., 2013) or without stirring (more simple–without stirring) (do Socorro Maia Quintino et al., 2002; Quintino and Angnes, 2004; Pereira et al., 2012; Tormin et al., 2014; Gimenes et al., 2015).

The use of BDD electrodes associated with BIA systems is recent. The first application was reported by Tormin et al. (2011). The BDD electrode was adapted to a home-made BIA cell with an internal volume of 180 mL (Figure 1). In this study, the phenolic antioxidant butylated hydroxyanisole (BHA) was determined in biodiesel samples using amperometric detection. A sample plug was directly injected on a cathodically pretreated BDD electrode that was immersed in 50% (v/v) hydroethanolic solution with 0.1 M HClO_4_. The proposed method presented a linear behavior from 10 to 50 μM for BHA determination and showed several advantages for routine biodiesel analysis, such as low relative standard deviation between injections (0.29%, *n* = 20), high analytical frequency (120 h^−1^), and simple sample treatment procedure (only dilution in electrolyte).

In the same year (2011), da Silva et al. (2011) showed the possibility of simultaneous determination using a single working electrode (BDD) and dual pulse amperometric detection (also called multiple pulse amperometry). The strategy was demonstrated using the following model analytes: (i) BHA and TBHQ (ii) paracetamol and caffeine, and (iii) dipyrone and caffeine. Limits of detection of 1.72, 0.72, 0.84, 0.19, and 0.04 μM were obtained for paracetamol, caffeine, dipyrone, TBHQ, and BHA, respectively. The analytical frequency of 60 injections per hour was achieved for all analysis.

In the following years, there was an increase in the number of papers which showed the use of BDD electrodes coupled to BIA systems for electrochemical detection of analytes in pharmaceutical samples. Cunha et al. (2013) reported an analytical method for determination of hydroquinone in pharmaceutical formulations employing amperometric detection and 0.1 M H_2_SO_4_ as the supporting electrolyte. A linear range from 10 to 2,000 μM, LOD of 0.016 μM, recovery values between 91 and 96% and an analytical frequency of 70 injections h^−1^ were achieved. The potential of BIA systems coupled to BDD electrodes and using multiple pulse amperometric (MPA) detection for simultaneous determinations was shown in the subsequent years. The BIA-MPA system was used successfully in the simultaneous determination of nimesulide and paracetamol (Pereira et al., 2013), codeine and diclofenac (Gimenes et al., 2013), propranolol and hydrochlorothiazide (Gimenes et al., 2014), diphenhydramine and 8-chlorotheophylline (dimenhydrinate) (Freitas et al., 2014), sulfamethoxazole and trimethoprim (Pereira et al., 2015), captopril and hydrochlorothiazide (Gimenes et al., 2015), amlodipine and atenolol (Silva et al., 2016), pheniramine and naphazoline (Oliveira et al., 2018b), or chlorpheniramine and naphazoline (Oliveira et al., 2018b). The possibility of selective determination of sildenafil (Garcia Cardozo et al., 2017) using the MPA technique was also recently shown. It is important to mention that all works employed a cathodically pretreated BDD electrode.

Concerning simultaneous determinations using BIA systems with multiple pulse amperometric detection, an improvement was shown by Freitas et al. (2016) with the simultaneous determination of three analytes (8-chlorotheophylline, pyridoxine, and diphenhydramine) in the same run using a single working electrode. In this work, a sequence of three potential pulses [+1.25, +1.60, and +1.80 V (vs. Ag/AgCl)] was applied with the acquisition of three separate amperograms. 8-Chlorotheophylline was detected selectively at +1.25 V, both 8-chlorotheophylline and pyridoxine at +1.60 V and 8-chlorotheophylline, pyridoxine, and diphenhydramine at +1.80 V. Subtraction between the currents detected at the three amperograms (with the aid of correction factors) was used for the selective determination of pyridoxine and diphenhydramine. According to the authors, the method presented advantages when compared to chromatographic separation systems, especially regarding to simplicity, cost-effectiveness, speed of analysis (60 injections h^−1^), and waste generation. Next, this system (BIA-MPA) was also used for the simultaneous determination of three species in pharmaceutical samples, such as phenazopyridine, sulfamethoxazole, and trimethoprim (Pereira et al., 2016); propyphenazone, paracetamol and caffeine (Silva et al., 2017b) and 8-chlorotheophylline, caffeine and diphenhydramine (Freitas et al., 2017a). All reported methods using the BIA-MPA system presented similar and relevant characteristics, such as fast analysis (≥60 injections h^−1^), minimal sample size requirements (≤150 μL), minimal waste production (≈150 μL by sample) usually without or with a minimal volume of organic solvent, possible implementation in laboratories with minimal infrastructure, and application to a wide range of active ingredients. Therefore, we can conclude that the combination of BIA-MPA method with to BDD electrodes presents numerous features for quality control of pharmaceutical samples.

Besides the analysis of pharmaceutical formulations, studies with other samples, such as insecticides and water, were also presented. Silva et al. (2014) described the determination of mancozeb in insecticide samples using BIA with pulsed amperometric detection (PAD). In this work, two potential pulses were optimized for detection and constantly electrochemical cleaning of the cathodically pre-treated BDD surface. A potential of +0.3 V was selected for anodic detection of mancozeb and as the cleaning potential, 0.0 V was used. The method provided good analytical characteristics, such as linear range of 40–650 μM, detection limit 0.5 μM and analytical frequency of 90 injections h^−1^. In other work, picoxystrobin was determined in natural water using a BIA with amperometric detection (Dornellas et al., 2015). The limit of detection and analytical frequency were 1.6 μM and 108 injections h^−1^, respectively. Afterwards, the liquid-liquid extraction method was associated with a BIA system with amperometric detection (Alencar et al., 2017). Very low concentrations levels (LOD = 0.08 μM) of levofloxacin were determined in tap, aquarium and lake water with a high analytical frequency (160 h^−1^).

The possibility of use of a fast voltammetric technique (square wave voltammetry) as detection mode in BIA flow systems coupled to BDD electrodes was also shown. Zinc and naphazoline were simultaneous determined in pharmaceutical samples by BIA with square wave voltammetric detection (Oliveira et al., 2016). The analysis was carried out in a single run which allowed the achievement of an elevated analytical frequency (70 injections h^−1^) and LODs of 0.13 and 0.4 μM for zinc and naphazoline, respectively. A portable screening method for cocaine as well as its main adulterants in seized cocaine samples was proposed by Freitas et al. (2017b) using BIA with square wave voltammetric detection and BDD as working electrode. In this study cocaine, benzocaine, caffeine, lidocaine, phenacetin, paracetamol, and procaine were selectively detected using BIA-SWV and BIA-MPA. A linear response was found for cocaine concentrations ranging from 6 to 30 ppm, with a detection limit of 0.27 ppm. The excellent selectivity that was achieved for cocaine detection was attributed to the use of an acidic medium (0.1 M H_2_SO_4_) as the electrolyte and BDD as the working electrode [oxidation of cocaine at potentials higher than +1.9 V (vs. Ag/AgCl)]. Recently, Silva et al. (2017a) showed the possibility of simultaneous determination of propyphenazone, paracetamol, and caffeine in pharmaceutical samples using BIA-SWV. In this work, the performance of a BIA-SWV system was compared with a conventional SWV system (steady state condition). According to the authors, the exchange and manipulation of the samples are simpler and faster with the BIA-SWV system (80 analysis h^−1^) in comparison to the conventional SWV system (20 analysis h^−1^). The most recent works employing BIA-SWV systems and BDD electrodes were focused on forensic chemistry. A new method for detection of adulterations with sibutramine in natural products and multivitamins supplements (Freitas et al., 2018), and a screening protocol for scopolamine in beverage and urine samples (Oliveira et al., 2018a) were presented. These papers introduce the new trends and perspectives about the wide range of applications of portable BIA systems employing electrochemical detection with BDD working electrodes. Table 2 presents a summary of the applications of BDD electrodes in BIA systems.

**Table 2:** BIA systems with electrochemical detection using BDD as the working electrode.

**DT**	**Pre-treatment**	**Analyte**	**Sample**	**LOD (μM)**	**LR (μM)**	**AF (h^**−1**^)**	**References**
DPA or MPA	−3 V/900 s/0.5 M H_2_SO_4_	TBHQ, BHA Paracetamol (PAR) Caffeine (CAF) Dipyrone (DYP)	Biodiesel and PF	1.7 (PAR) 0.7 (CAF) 0.8 (DYP) 0.2 (TBHQ) 0.04 (BHA)	NR	60	da Silva et al., 2011
AMP	−3 V/900 s/0.5 M H_2_SO_4_	BHA	Biodiesel	0.05	10–50	120	Tormin et al., 2011
AMP	−3 V/900 s/0.2 M H_2_SO_4_	Hydroquinone	PF	0.02	10–2000	70	Cunha et al., 2013
MPA	−0.01 A/1,000 s/0.1 M H_2_SO_4_	Codeine (COD) Diclofenac (DCF)	PF	1.0 (COD) 1.1 (DCF)	7–36 (COD) 10–50 (DCF)	300	Gimenes et al., 2013
MPA	−0.01 A/1,000 s/0.1 M H_2_SO_4_	Nimesulide (NIM) Paracetamol (PAR)	PF	0.3 (PAR) 0.3 (NIM)	10–50 (NIM) 50–250 (PAR)l	46	Pereira et al., 2013
MPA	−0.01 A/1,000 s/0.1 M H_2_SO_4_	Propranolol (PRO) Hydrochlorothiazide (HZT)	PF	0.2 (PRO) 1.9 (HZT)	10–50 (PRO) 5–26 (HZT)	130	Gimenes et al., 2014
MPA	−0.01 A/1,000 s/0.1 M H_2_SO_4_	Diphenhydramine (DIP) 8-chlorotheophylline (8-CTP)	PF	0.1 (8-CTP) 0.2 (DIP)	10–80 (8-CTP) 10–60 (DIP)	70	Freitas et al., 2014
PAD	−0.01 A/1,000 s/0.1 M H_2_SO_4_	Mancozeb	Insecticide	0.5	40–650	90	Silva et al., 2014
MPA	−0.01 A/1,000 s/0.1 M H_2_SO_4_	Picoxystrobin	Natural water	1.6	5–100	108	Dornellas et al., 2015
MPA	−0.01 A/1,000 s/0.1 M H_2_SO_4_	Sulfamethoxazole (SMX) and trimethoprim (TMP)	PF	0.9 (SMX) 0.6 (TMP)	40–198 (SMX) 7–35 (TMP)	75	Pereira et al., 2015
MPA	−0.01 A/1,000 s/0.1 M H_2_SO_4_	Captopril (CAP) Hydrochlorothiazide (HZT)	PF	0.1 (CAP) 0.3 (HZT)	27–81 (CAP) 10–30 (HZT)	100	Gimenes et al., 2015
MPA	−0.01 A/1,000 s/0.1 M H_2_SO_4_	8-chlorotheophylline (8-CTP) Pyridoxine (PYR) Diphenhydramine (DIP)	PF	0.2 (8-CTP) 0.5 (PYR) 0.2 (DIP)	10–100 (8-CTP and DIP) 10–60 (PYR)	60	Freitas et al., 2016
SWV and SWASV	−0.01 A/1,000 s/0.1 M H_2_SO_4_	Zinc Naphazoline (NAP)	PF	0.1 (Zn^2+^) 0.04 (NAP)	3–17 (NAP) 10–50 (Zn^2+^)	70	Oliveira et al., 2016
MPA	−0.01 A/1,000 s/0.1 M H_2_SO_4_	Phenazopyridine (PHE) Sulfamethoxazole (STZ) Trimethoprim (TRI)	PF	0.5 (PHE) 0.8 (STZ) 0.7 (TRI)	18–1,500 (STZ) 8–158 (TRI) 3–138 (PHE)	70	Pereira et al., 2016
PAD	−0.01 A/1,000 s/0.1 M H_2_SO_4_	Amlodipine (AML) Atenolol (ATL)	PF	0.07 (AML) 0.07 (ATL)	2–100 (AML) 2–75 (ATL)	70	Silva et al., 2016
SWV and MPA	−0.01 A/1,000 s/0.1 M H_2_SO_4_	Cocaine (COC)	Seized cocaine	0.9 (COC)	20–100 (COC)	NR	Freitas et al., 2017b
MPA and SWV/PCA	−0.01 A/1,000 s/0.1 M H_2_SO_4_	Sildenafil	PF	N.R.	5–150	60	Garcia Cardozo et al., 2017
MPA	−0.01 A/1,000 s/0.1 M H_2_SO_4_	Paracetamol (PAR) Propyphenazone (PRO) Caffeine (CAF)	PF	1.3 (PAR) 1.3 (PRO) 0.5 (CAF)	7–562 (PAR) 22–217 (PRO) 10–82 (CAF)	75	Silva et al., 2017a
MPA	−0.01 A/1,000 s/0.1 M H_2_SO_4_	8-chlorotheophylline (8-CTP) Caffeine (CAF) Diphenhydramine (DIP)	PF	0.3 (8-CTP) 0.5 (CAF) 0.8 (DIP)	10–100 (8-CTP and DIP) 10–140 (CAF)	120	Freitas et al., 2017a
AMP	−0.01 A/1,000 s/0.1 M H_2_SO_4_	Levofloxacin	Tap, aquarium and lake water	0.1	5–100	160	Alencar et al., 2017
SWV	−0.01 A/1,000 s/0.1 M H_2_SO_4_	Propyphenazone (PRO) Paracetamol (PAR) Caffeine (CAF)	PF	0.9 (PRO) 2.0 (PAR) 1.5 (CAF)	9–217 (PRO) 13–331 (PAR) 10–257 (CAF)	80	Silva et al., 2017b
MPA	−0.01 A/1,000 s/0.1 M H_2_SO_4_	Pheniramine (PHE) Chlorpheniramine (CHL) Naphazoline (NAP)	PF	0.6 (PHE) 0.5 (CHL) 0.1 (NAP)	16–120 (CHL and PHE) 2–15 (NAP)	80	Oliveira et al., 2018b
BIA–SWV	−0.01 A/1,000 s/0.1 M H_2_SO_4_	Sibutramine	Natural products and multivitamins	0.3	18–180	NR	Freitas et al., 2018
BIA–SWV	+0.01 A/1,000 s/0.12 M Britton-Robinson buffer	Scopolamine	Beverage and Urine Samples	0.2	1–20	NR	Oliveira et al., 2018a

*DT, detection technique; DPA, dual pulse amperometry; MPA, multiple pulse amperometry; AMP, amperometry; PAD, pulsed amperometric detection; SWV, square-wave voltammetry; SWASV, square-wave anodic stripping voltammetry; PF, pharmaceutical formulation; LOD, limit of detection; LR, linear range; AF, analytical frequency; NR, not reported*.

## BDD Electrode Coupled to HPLC Systems

The electrochemical detector is one of the most sensitive and selective HPLC detectors available for analytes that undergo oxidation or reduction reactions (Swartz, 2010). The use of BDD electrodes for electrochemical detection in HPLC systems started in 2000 (Rao et al., 2000). In this study sulfadiazine, sulfamerazine, and sulfamethazine were detected at an applied potential of +1.0 V vs. Ag/AgCl. Both GCE and BDD electrodes exhibited similar detection limits, however, faster background current stabilization was observed for the BDD (10 min) electrode at +1.0 V vs. Ag/AgCl, whereas GCE required more than 30 min. In addition, the performance of BDD was stable for several hours without requiring chemical or mechanical pretreatment.

Anodized BDD electrodes were majorly employed in the first applications of electrochemical detection in HPLC systems (Chailapakul et al., 2002; Terashima et al., 2002; Charoenraks et al., 2005; Bouvrette et al., 2006; Ivandini et al., 2007; Andrade et al., 2009). Terashima et al. (2002) presented the use of anodically pretreated BDD electrodes for the detection of chlorophenols in environmental water samples (condensed from the flue gas of waste incinerators). The performance of the BDD electrode was better than the obtained with GCE due to its resistance to fouling even in the presence of high concentrations of chlorophenols. This phenomenon was explained by the anodically generated oxygen functional groups on the BDD surface that repel phenoxy radicals, which are responsible for the formation of polymeric films that passivates the electrode surface. In another work from the same research group (Chailapakul et al., 2002), homocysteine was detected with high sensitivity in acidic media using amperometric detection on the BDD electrode. Excellent analytical characteristics were achieved, such as a detection limit of 1 nM, response variability lower than 1% (*n* = 9) and a linear dynamic range from 0.005 to 100 μM.

BDD electrodes were also used in different applications in HPLC systems without electrochemical pretreatment. Ivandini et al. (2002) only pretreated the electrode by ultrasonication in 2-propanol for about 10 min followed by rinsing with high purity water with the purpose to remove possible remaining organic impurities originated during the deposition of diamond in the manufacture of the electrode. The electrode was employed to the electrochemical detection of six antidepressant drugs in blood; LODs in nanomolar levels were achieved. Rao et al. (2002) presented a study for determination of carbamates pesticides using an as-deposited electrode. After a prolonged use, or in case of fouling of the electrode, an on-line reactivation by anodic treatment at ~3 V (vs. SCE) for 30 min was performed. Detection limits between 0.6 and 1 ppb were achieved, comparable with well-established detection methods as HPLC-fluorescence. Afterwards, the possibility of detecting of species of environmental (Treetepvijit et al., 2006; Pecková et al., 2009), pharmaceutical (Siangproh et al., 2010; Maixnerová et al., 2012; Mahé et al., 2015; Long et al., 2017) and biological (Bartošová et al., 2011; Kotani et al., 2011; Zhang et al., 2016) interests using a BDD electrode without electrochemical pretreatment coupled to HPLC systems have been also presented.

The use of cathodically pretreated BDD electrodes coupled to HPLC systems was presented for the first time by Martins et al. (2010, 2011). In this work, prior the first use, an anodic treatment (3.0 V (vs. Ag/AgCl) applied for 10 min) was done followed by a cathodic polarization for 10 min at −3.0 V. Pulsed amperometry was employed to the detection of benzodiazepines in pharmaceutical samples by reduction at −1.9 V in a thin layer mode cell. Detection limits from 0.5 to 2 ppm were reached. The same treatment was performed for the determination of three different parabens in shampoo samples. The anodic detection was performed at +1.2 V (vs. Ag/AgCl) with pulsed amperometric detection. In addition to the pretreatment, a cathodic on-line treatment (−3 V/30 s) was used to restore the electrode surface in case of fouling of the BDD electrode. The use of such high potentials is only possible with BDD electrodes. An alternative process for cathodic pretreatment of the electrode was employed by de Amorim and Andrade (de Amorim and Andrade, 2017) consisting of an anodic polarization (0.5 A cm^−2^ for 30 s) followed by a cathodic pretreatment (−0.5 A cm^−2^ for 150 s) in a 0.5 M H_2_SO_4_ solution for estrogens detection. Estrone and estradiol were determined after extraction from human urine. Limits of detection of 57 and 53 ppb for estrone and estradiol were obtained, respectively. Table 3 shows a summary of the use of BDD electrodes in the electrochemical detection of HPLC systems.

**Table 3:** HPLC systems with electrochemical detection using BDD as the working electrode.

**DT**	**Pre-treatment**	**Analyte**	**Sample**	**LOD (μM)**	**LR (μM)**	**AF**	**References**
AMP	As received	Sulfadiazine, sulfamerazine, and sulfamethazine	NR	0.05	0.05–50 for all	9	Rao et al., 2000
AMP	+2.64 V/4 min/Britton-Robinson Buffer–pH 2	Chlorophenols	Drainwater	0.02	20–100 for all	2	Terashima et al., 2002
AMP	As received	Imipramine (IM) Desipramine (DE) Clomipramine (CL) Amitriptyline (AM) Nortriptyline (NO) Doxepin (DO)	Blood	0.003 (IM and DE) 0.0005 (CL) 0.163 (AM) 1.08 (NO) 0.09 (DO)	0.05–100	3	Ivandini et al., 2002
AMP	+2.4 V/30 min/0.1 M KOH	Homocysteine	–	0.001	0.05–100	4	Chailapakul et al., 2002
AMP	As received	Carbofuran (CAN) Bendiocarb (BEN) Carbaryl (CAL) Dichloron (DIC)	Pesticides	0.06 (CAR) 0.10 (BEN) 0.10 (CAL) 0.03 (DIC)	0.10–100	6	Rao et al., 2002
PAD	Cyclic voltammetry (0–2 V; 10 mV/s)/30 min/0.1 M KOH	Oxytetracyline (OX) Tetracycline (TE) Chlortetracycline (CT) Doxycycline (DO)	Shrimp	0.1 (OX) 0.1 (TE) 0.2 (CT) 0.2 (DO)	0.2–217 (OX) 0.2–225 (TE) 0.2–209 (CT) 0.2–225 (DO)	2	Charoenraks et al., 2005
AMP	+2.5 V/10 min/mobile phase	16 polycyclic aromatic hydrocarbons	NR.	0.01–0.04	0.025–50 (depending on each analyte)	4	Bouvrette et al., 2006
AMP	As received	Oxytetracyline (OX) Tetracycline (TE) Chlortetracycline (CT) Doxycycline (DO)	Shrimp	0.02 (OX) 0.02 (TE) 0.1 (CT) 0.1 (DO)	0.1–217 (OX) 0.1–225 (TE) 0.2–209 (CT) 0.2–225 (DO)	2	Treetepvijit et al., 2006
AMP	+3.0 V/20 min/Britton Robinson buffer–pH 2.1	Purine and pyrimidine	DNA	0.03–0.16	0.1–10 for all	4	Ivandini et al., 2007
AMP	+0.5 A cm^−2^/60 s/0.5 M H_2_SO_4_	Sulfamethoxazole (SFX) Trimethoprim (TRM)	Bovine milk	0.1 (SFX) 0.05 ppb (TRM)	0.2–3.2 (SFX) 0.09–1.38 (TRM)	2	Andrade et al., 2009
AMP	As received	Amino derivatives of biphenyl	Drinking and river water	0.1 for all	0.1–10 for all	10	Pecková et al., 2009
AMP	−3.0 V/10 min/NR	Nitrazepan (NIT) Clonazepan (CLO) Diazepan (DIA)	Pharmaceutical	1.8 (NIT) 1.9 (CLO) 7.0 (DIA)	NR	3	Martins et al., 2010
AMP	As received	α-lipoic acid	Dietary supplement	0.01	0.05–291	10	Siangproh et al., 2010
PAD	−3.0 V/10 min/NR	Methylparaben (MePa) Ethylparaben (EtPa) Propylparaben (PrPa)	Shampoo	2.6 (MePa) 2.4 (EtPa) 2.2 (PrPa)	3.3–131 (MePa) 3.0–120 (EtPa) 2.8–111 (PrPa)	7.5	Martins et al., 2011
AMP	NR	Sildenafil (SL), vardenafil (VR), and their main metabolites	Human plasma	0.006 (SL) 0.006 (VR)	0.02–0.84 (SL) 0.02–0.82 (VR)	5	Bartošová et al., 2011
AMP	As received	Cholesterol	Meat	0.008	0.02–100 μM	1	Kotani et al., 2011
AMP	NR	Aminobiphenyls	Synthetic colorant tartrazine	0.07 (thin-layer) 0.02 (wall-jet)	0.2–10 (thin-layer) 0.06–100 (wall-jet)	10	Maixnerová et al., 2012
AMP	NR	Hydroquinone	–	NR	NR	NR	Mahé et al., 2015
AMP	NR	Eleven bioamines	Cortex and hippocampus rats	0.01–0.44	0.05–331 (depending on each analyte)	2	Zhang et al., 2016
AMP	+0.5 A cm^−2^/30 s/0.5 M H_2_SO_4_	Estrone (ETE) Estradiol (ETL)	Human urine	0.21 (ETE) 0.19 (ETL)	NR	4	de Amorim and Andrade, 2017
AMP	NR	Cyclovirobuxin D	Tablets and human blood	0.0005	0.0007–4.7	2	Long et al., 2017

*DT, detection technique; LR, linear range; AF, analytical frequency; AMP, amperometry; PAD, pulsed amperometric detection; LOD, limit of detection; NR, not reported*.

## BDD Electrode Coupled to CE Systems

The BDD electrode was coupled with conventional CE for the first time by Shin et al. (2003) for end-column amperometric detection of dopamine (DP), norepinephrine (NOR), and epinephrine (EPI). Low detection limits (19 nM for EPI) and highly reproducible current responses (RSD < 5%, *n* = 10) were obtained for successive injections of a solution containing 50 μM DP, NOR, and EPI. The potentiality of the BDD electrode for the determination of phenol and five chlorinated phenols was also shown.

In the year of 2003, the Swain's group (Cvačka et al., 2003) report the fabrication and characterization of diamond microelectrodes formed on sharpened platinum wires. According to the authors, the response of these microelectrodes was affected by the boron-doping level (ohmic resistance) and shape of the electrode, and they exhibited reproducible voltammetric responses, at least in terms of the peak and half-wave potentials for a variety of aqueous redox analytes. In addition, the basic electrochemical properties of these diamond microelectrodes (low background and noise currents, reproducible responsiveness, wide linear dynamic range and low limits of detection) demonstrated the usefulness of this new electrode material for electrochemical detection in CE. Afterwards, the performance of these diamond microelectrodes was shown in the detection of dopamine and catechol; (ii) chlorinated phenols in natural waters (Muna et al., 2005), endogenous norepinephrine in sympathetic nervous system of rats (Park et al., 2006), and endogenous norepinephrine from spleen, small intestine, and heart of normotensive rats (Quaiserová-Mocko et al., 2008). Some figures of merit for the aforementioned works can be found in Table 3 (BDD electrodes and HPLC systems).

BDD electrodes were also coupled with CE microchips. The performance of BDD electrodes as end-column amperometric detector of microchip CE systems was first demonstrated by Wang's group (Wang et al., 2003). Good sensitivity, lower noise levels, and sharper peaks were obtained for different groups of analytes (nitroaromatic explosives, organophosphate nerve agents, and phenols) when compared to screen-printed carbon electrode. One of the advantages related by the authors is the reduced surface fouling offered by the BDD electrode (RSD = 0.8%, *n* = 60) in contrast with the screen-printed carbon electrode (RSD = 10.8%). The limits of detection of 1,3-dinitrobenzene and 2,4-dinitrotoluene were 70 and 110 ppb, respectively. In a subsequent study, the same research group (Wang et al., 2004) performed the separation and detection of five purines and purine-containing compounds (guanine, hypoxanthine, guanosine, xanthine, and uric acid). In this work, fast analyses were possible (6 min) using a low separation voltage (+1,000 V), if compared to conventional CE. Linear coefficients (≥0.998) and acceptable limits of detection (≤2.1 μM) were obtained. The characteristics of the BBD electrode was also explored for the detection of several amino-substituted aromatic compounds (4-aminophenol, 1,2-phenylenediamine, 2-aminonaphthalene, 2-chloroaniline, and o-aminobenzoic acid) using a CE microchip (Shin et al., 2004). In this work, the BDD electrode (2.4%) showed smaller decrease in the detected current as function of time than screen-printed (21.8%) and glassy-carbon (28.3%) electrodes. Wide linear response ranges (2–50 μM) and low limits of detection (<2.0 μM) were obtained for all target compounds. A summary of the use of BDD electrodes coupled to CE systems can be seen in Table 4.

**Table 4:** Conventional and Microchip CE systems with electrochemical detection using BDD as the working electrode.

**DT**	**Pre-treatment**	**Analyte**	**Sample**	**LOD (μM)**	**LR (μM)**	**AF**	**REF**
**CONVENTIONAL CE SYSTEM**							
AMP	NR	Dopamine (DP) Norepinephire (NOR) Epinephrine (EPI)	NR	0.02 (DP) 0.02 (NOR) 0.02 (EPI)	0.1–100	3	Shin et al., 2003
AMP	Water rinsed	Catechol (CTC) Dopamine (DOP)	Standard solution	0.1 (CTC) 0.08 (DOP)	0.1–100 (CTC) 0.08–100 (DOP)	7	Cvačka et al., 2003
AMP	NR	2-Chlorophenol (2-CP) 3-Chlorophenol (3-CP) 4-Chlorophenol (4-CP) 2,4-Dichlorophenol (2,4-DCP) 2,4,6-Trichlorophenol (2,4,6-TCP) Pentachlorophenol (PCP)	Natural water	1.6 × 10^−4^ (2-CP) 1.6 × 10^−4^ (3-CP) 1.6 × 10^−4^ (4-CP) 2.4 × 10^−4^ (2,4-DCP) 1.0 × 10^−3^ (2.4.6-TCP) 1.9 × 10^−4^ (PCP)	1.6 × 10^−4^ −0.78 (2-CP/3-CP/4-CP) 2.4 × 10^−4^ −0.80 (2,4-DCP) 1.0 × 10^−3^ −0.76 (2,4,6-TCP) 1.9 × 10^−4^ −0.75 (PCP)	4	Muna et al., 2005
AMP	Rinsing/isopropyl alcohol	Dopamine hydrochloride (DA) Norepinephrine (NE) DL-Normetanephrine hydrochloride (NM) DL-3,4-Dihydroxyphenylethyleneglycol (DOPEG) DL-Vanillyl-mandelic acid (VMA)	Standard solution	0.04 (DA) 0.05 (NE) 0.04 (NM) 0.25 (DOPEG) 0.15 (VMA)	0.05–100 (DA/NE/NM) 0.25–50 (DOPEG) 0.100–50 (VMA)	4	Park et al., 2006
AMP	NR	Norepinephrine (NE)	Biological tissue	0.05	0.08–1.00	9	Quaiserová-Mocko et al., 2008
**MICROCHIP CE SYSTEM**							
AMP	Sonication/2-propanol/10 min.	1,3-Dinitrobenzene (1,3-DNB) 2,4-Dinitrotoluene (2,4-DNT)	NR	0.42 (1,3 DNB) 0.60 (2,4-DNT)	1.2–8.3 (1,3 DNB) 1.1–7.7 (2,4-DNT)	20	Wang et al., 2003
AMP	Sonication/2-propanol/10 min.	Guanosine (GNS) Xanthine (XNT)	NR	2.2 (GNS) 2.1 (XNT)	50–250	18	Wang et al., 2004
AMP	Sonication/2-propanol/10 min.	4-Aminophenol (4-AP) 2-Aminonaphthalene (2-AN)	NR	2.0 (4-AP) 1.3 (2-AN)	2–50	36	Shin et al., 2004

*DT, detection technique; AF, analytical frequency; LR, linear range; AMP, amperometry; PAD, pulsed amperometric detection; LOD, limit of detection; NR, not reported*.

## Concluding Remarks

This review provided an overview on the use of BDD electrodes for electrochemical detection in flow systems (FIA, BIA, HPLC, and CE). The highly favorable properties of BDD electrodes enabled their successful application in electroanalysis for the development of analytical methods with long-term response stability, wide linear dynamic ranges and low limits of detection. The synergistic effect between BDD electrodes and flow systems is especially observed in the detection of analytes that require high positive potentials. If solid electrodes are used, the BDD material presents the best response stability and thus high-throughput methods can be developed. Particular attention should be given to the type of applied electrochemical pretreatment (cathodic and/or anodic) carried out before the analyses, because the electroanalytical performance of the BBD electrode can be highly influenced by this process. Therefore, the ideal protocol to work with BDD electrodes for flow analysis is to design a flow-cell that enables the electrochemical pretreatment step and detection sequentially to facilitate routine applications and on-site measurements.

## Data Availability

All datasets generated for this study are included in the manuscript and/or the Supplementary Files.

## Author Contributions

Contributions of ER and RM were planning and writing of the review paper. Contributions of JF and TO are literature review, collection, writing, editing, and format arrangement.

### Conflict of Interest Statement

The authors declare that the research was conducted in the absence of any commercial or financial relationships that could be construed as a potential conflict of interest.

## Abbreviations

BDD: boron-doped diamond
FIA: flow injection analysis
BIA: batch injection analysis
CE: capillary electrophoresis
HPLC: high performance liquid chromatography
GCE: glassy carbon electrode
AMP: amperometry
MPA: multiple pulse amperometric.
